# Vitamin C Status in People with Types 1 and 2 Diabetes Mellitus and Varying Degrees of Renal Dysfunction: Relationship to Body Weight

**DOI:** 10.3390/antiox11020245

**Published:** 2022-01-27

**Authors:** Anitra C. Carr, Emma Spencer, Helen Heenan, Helen Lunt, Monica Vollebregt, Timothy C. R. Prickett

**Affiliations:** 1 Nutrition in Medicine Research Group, Department of Pathology and Biomedical Science, University of Otago, Christchurch 8011, New Zealand; emma.spencer@otago.ac.nz; 2 Diabetes Outpatients, Canterbury District Health Board, Christchurch 8011, New Zealand; helen.heenan@cdhb.health.nz (H.H.); helen.lunt@cdhb.health.nz (H.L.); 3 Department of Medicine, University of Otago, Christchurch 8011, New Zealand; tim.prickett@otago.ac.nz; 4 Centre for Postgraduate Nursing Studies, University of Otago, Christchurch 8011, New Zealand; volmo510@student.otago.ac.nz

**Keywords:** vitamin C, ascorbate, diabetes, type 1 diabetes, type 2 diabetes, renal dysfunction, hypertension, body weight, BMI

## Abstract

Diabetes mellitus is a chronic metabolic disorder and is associated with depleted vitamin C status. The underlying aetiologies and pathogeneses responsible for this association are poorly understood. This retrospective study explored the vitamin C status of 136 adult outpatients with types 1 and 2 diabetes mellitus (T1DM/T2DM), with a focus on indices of renal function and metabolic health, including body weight. In the T1DM group (*n* = 73), the median plasma vitamin C concentration was 33 (18, 48) µmol/L, with 37% hypovitaminosis C and 12% deficiency. In the T2DM group (*n* = 63), the median plasma concentration was 15 (7, 29) µmol/L, with 68% hypovitaminosis C and 38% deficiency. Lower vitamin C was associated with macroalbuminuria (*p* = 0.03), renal dysfunction (*p* = 0.08), and hypertension (*p* = 0.0005). Inverse associations were also observed between plasma vitamin C and various other metabolic health parameters (*p* < 0.05), especially body weight (*p* < 0.0001), which was higher in those with hypovitaminosis C (<23 µmol/L; *p* = 0.0001). The association with bodyweight remained, even after multivariable analysis. In summary, body weight was a significant predictor of low vitamin C status in people with diabetes. This suggests that people with both diabetes and a high body weight may have greater than average vitamin C requirements.

## 1. Introduction

Diabetes mellitus is a complex disorder characterised by chronic metabolic dysregulation and potentially life-threatening complications [1]. Type 1 diabetes mellitus (T1DM) results from a deficiency of insulin secretion and has genetic and autoimmune risk factors. The more common type 2 diabetes mellitus (T2DM) results from resistance to insulin action and may be present for many years before detection. Chronic hyperglycaemia can result in severe complications such as nephropathy, retinopathy, neuropathy, and cardiovascular diseases [1]. In 2019, worldwide cases of diabetes comprised approximately 463 million people, with approximately 38 million new cases of diabetes since 2017 [2,3,4]. Significant socioeconomic and ethnic disparities are observed in many regions of the world, including in Australia and New Zealand, where indigenous Aboriginal, Māori and Pasifika peoples have a higher prevalence of T2DM and its associated complications [5,6].

Obesity is a major risk factor for diabetes, and research has indicated that the chronic low-grade inflammation and oxidative stress commonly observed in people with obesity and diabetes play a pivotal role in the development and progression of the disease [7,8]. Oxidative stress is characterised by an imbalance in the generation of reactive oxygen species in the body and the ability of endogenous antioxidant systems to neutralise these. Vitamin C is an essential micronutrient with potent antioxidant properties [9]. Epidemiological evidence indicates that higher plasma vitamin C concentrations are associated with decreased risk of developing T2DM [10,11]. Vitamin C is able to scavenge a wide range of reactive oxygen species, thereby protecting essential biomolecules from oxidative damage [12]. We and others have previously shown lower vitamin C status and a higher prevalence of hypovitaminosis C and outright deficiency in people with T2DM, despite comparable dietary intakes of the vitamin to healthy controls [13,14]. 

Of note, obesity was an independent predictor of vitamin C status in these cohorts. This finding suggests that the elevated inflammation and oxidative stress observed in people with diabetes and obesity may be depleting their vitamin C concentrations independent of dietary intake. Intestinal inflammation may also attenuate uptake of dietary vitamin C via the intestinal vitamin C transporter (SVCT1) [15]. Furthermore, there may be a volumetric dilution effect in people with higher body weight. This premise is supported by a supplementation study indicating lower plasma vitamin C concentrations in people with higher body weight, despite comparable vitamin C intakes [16]. Other research has suggested that lower vitamin C status in patients with both T1DM and T2DM may be due to enhanced renal excretion in those with evidence of underlying renal disease [17,18].

A number of studies assessing vitamin C status in people with diabetes have included both T1DM and T2DM [19,20,21,22]; however, fewer studies have directly compared the vitamin C status of people with T2DM relative to those with T1DM [21,22]. Some have found lower vitamin C status in people with T2DM relative to those with T1DM [22], whilst others have found comparable vitamin C status between the two types [21]. The primary aim of the current study was to explore the associations between plasma vitamin C and markers of renal and cardiometabolic health, with a focus on obesity, in people with diabetes. A secondary aim was to provide a descriptive comparison of these markers by diabetes diagnosis (T1DM or T2DM), noting that there are inherent cardiometabolic difference between these two types of diabetes.

## 2. Methods

### 2.1. Study Participants

This study was a retrospective analysis of blood samples collected for a study on markers of chronic kidney disease in patients with either T1DM or T2DM [23]. The participants were recruited from a single outpatient diabetes clinic at Christchurch Hospital, New Zealand (between June 2017 and June 2018). Diabetes diagnosis (T1DM versus T2DM) was made by the attending diabetes specialist based on clinical phenotype, plus additional laboratory tests as required. The patients were recruited to ensure that a broad range of renal function (as determined by estimated glomerular filtration rate; eGFR, and albuminuria, i.e., albumin-to-creatinine ratio; ACR) were equivalently represented in both T1DM and T2DM groups [23]. eGFR was calculated using the CKD-EPI creatinine equation [24]. Exclusions were: aged >80 years, previous bariatric surgery and any life-threatening comorbidity. For T1DM, volunteers aged <25 years and those where duration was <5 years were excluded; for T2DM, only those aged >30 years were invited to participate. Thus, the study cohort was not representative of the general diabetes population. The participants underwent a review of personal physical history and anthropometric measures (height, weight, blood pressure), as well as a questionnaire about their general health. Non-fasting blood samples were collected, and following rapid processing at 4 °C, the plasma was stored at −80 °C for subsequent analyses.

Ethical approval for analysis of the blood samples for the current study was obtained from the New Zealand Northern B Health and Disabilities Ethics Committee (#19/NTB/207). Participants from the earlier study were re-contacted by a research nurse to ascertain whether they would consent to their stored plasma samples being analysed for vitamin C content prior to disposal of the samples. Of the original cohort of 202 participants, 138 consented, 2 did not consent, 49 did not respond and 13 were deceased. No attempt was made to contact the families of the deceased patients to seek permission for sample analysis. Of the 138 who consented, blood samples were not available for two of these patients, resulting in a final cohort of 136 patients. Demographic information (age, sex, ethnicity) was collated, and New Zealand deprivation index (NZDep2013) was determined from the domicile code of the participants’ addresses.

### 2.2. Plasma Vitamin C and Biomarker Analysis

For vitamin C analysis, the plasma samples were treated with an equal volume of ice-cold perchloric acid (PCA; 0.54 M) solution containing the metal chelator diethylenetriaminepentaacetic acid (DTPA; 100 µmol/L) to precipitate proteins and stabilise the vitamin C. To recover any vitamin C that had become oxidised during processing and/or storage, the PCA-supernatant was treated with 10% *v/v* tris(2carboxyethyl)phosphine (TCEP; 350 mmol/L) for 3 h at 4 °C. The treated supernatant was then analysed for total vitamin C content using reverse-phase high-performance liquid chromatography (HPLC) with electrochemical detection, as previously described [25]. Vitamin C category cut-off values were defined as: deficient ≤11 µmol/L; hypovitaminosis C ≤23 µmol/L; inadequate <50 µmol/L; adequate ≥50 µmol/L; saturating ≥70 µmol/L [26].

Standard renal function parameters (i.e., plasma cystatin C, serum and urine creatinine, urine albumin, ACR, eGFR) and metabolic health markers (i.e., triglycerides, total cholesterol, HDL cholesterol, LDL cholesterol, urate and HbA1c) were measured at Canterbury Health Laboratories, an International Accreditation New Zealand (IANZ) laboratory as part of the original study [23].

### 2.3. Statistical Analysis

Data are presented as median and interquartile range (Q1, Q3) and normality was tested using the Shapiro–Wilk test. Regression analyses were carried out using Spearman correlation and differences between groups were determined by Mann–Whitney U tests or analysis of variance (ANOVA), with *p* < 0.05 signifying significance. Statistical analyses were carried out using GraphPad Prism 9 (GraphPad Software, San Diego, CA, USA). Linear multivariable regression was undertaken using R 4.1.1 to investigate the association between plasma vitamin C (dependent variable) and diabetes type (independent variable) after adjusting for various potentially confounding variables (weight, age, gender, diastolic blood pressure, cholesterol to HDL ratio, HbA1c, ACR) [27].

## 3. Results

### 3.1. Participant Characteristics

The total cohort of patients with diabetes (*n* = 136) comprised 73 (54%) patients with T1DM and 63 (46%) patients with T2DM. The median age for the total cohort was 57 (44, 67) years; the patients with T2DM were older and of shorter duration of known diabetes than the patients with T1DM (Table 1). Of the total participants, 66 (44%) had renal dysfunction (defined as eGFR-EPI < 60 mL/min/1.73 m^2^ or ACR > 3 g/mol); the T1DM and T2DM groups had been recruited to have a comparable proportion of renal dysfunction, although the patients with T2DM had more severe renal dysfunction (as evidenced by differences in renal function parameters (cystatin C, ACR and eGFR; Table 2). The patients with T2DM had higher weight, BMI and blood pressure than the patients with T1DM (Table 2).

### 3.2. Vitamin C Status in People with T1DM and T2DM

**Figure 1:**
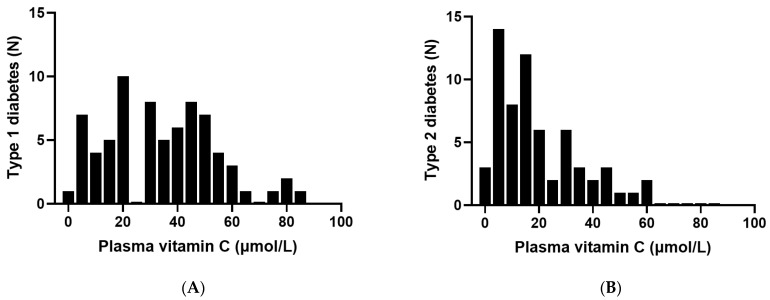
Frequency distribution of plasma vitamin C in patients with diabetes: (**A**) type 1 diabetes (*n* = 73) and (**B**) type 2 diabetes (*n* = 63).

**Figure 2:**
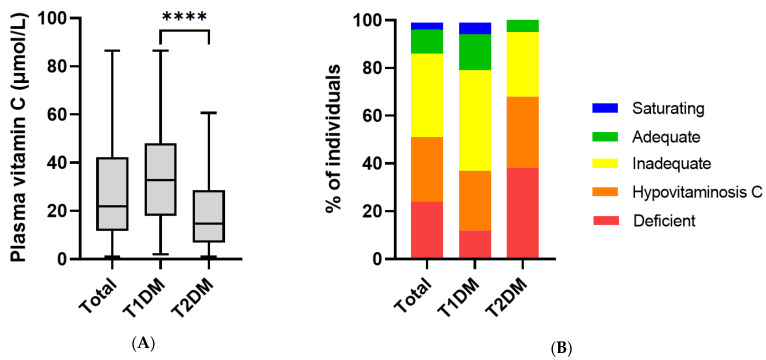
Vitamin C status in patients with T1DM and T2DM: (**A**) box plots show median, borders the 25th and 75th percentiles and whiskers the range; **** *p* < 0.0001 (Mann–Whitney U test). (**B**) Vitamin C categories: deficient ≤ 11 µmol/L; hypovitaminosis C ≤ 23 µmol/L; inadequate < 50 µmol/L; adequate ≥ 50 µmol/L; saturating ≥ 70 µmol/L.

Five participants (3.7% of the cohort) were taking vitamin supplements that contained low, dietary level doses (15–100 mg) of vitamin C. These were predominantly patients with T2DM and were included in all analyses. Frequency distribution of the patients studied indicated a normal distribution for the T1DM group and a skewed distribution for the T2DM group, with a high proportion of patients with T2DM having low vitamin C status (Figure 1). The vitamin C status of the total cohort was 22 (12, 42) µmol/L. The participants with T1DM had a median vitamin C concentration of 33 (18, 48) µmol/L, and those with T2DM had 15 (7, 29) µmol/L, *p* < 0.0001; Figure 2A). The patients with T2DM had a high proportion of insufficiency (95% < 50 µmol/L), hypovitaminosis C (68% ≤ 23 µmol/L) and deficiency (38% ≤ 11 µmol/L). The patients with T1DM had 79% insufficiency, 37% hypovitaminosis C, 12% deficiency (Figure 2B).

### 3.3. Plasma Vitamin C Status Relative to Renal Function Parameters

**Figure 3:**
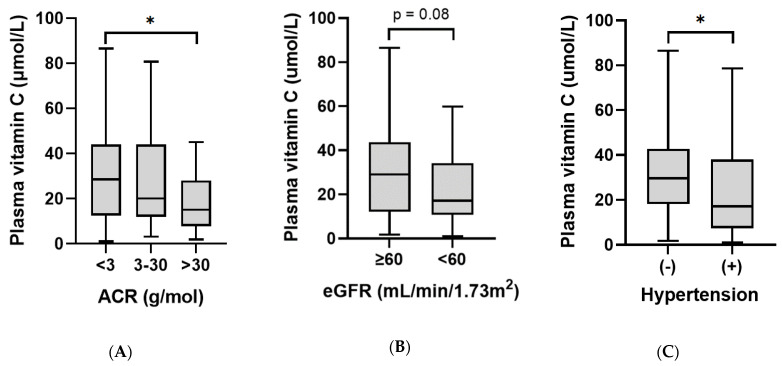
Plasma vitamin C relative to renal function and hypertension: (**A**) patients with macroalbuminuria (ACR > 30) had significantly lower plasma vitamin C concentrations than those with normal renal function (ACR < 3; * *p* = 0.03). (**B**) Patients with eGFR <60 had comparable plasma vitamin C concentrations to those with eGFR ≥ 60 (*p* = 0.08). (**C**) Patients with treated hypertension (+) had lower vitamin C status than those without (−) (* *p* = 0.01).

Because vitamin C status is regulated by renal excretion and tubular reabsorption of the vitamin, we assessed vitamin C status relative to renal function parameters. In the whole cohort, there was a weak inverse association between vitamin C status and ACR (*r* = −0.21, *p* = 0.01; Table 3). Renal dysfunction was categorised using ACR cut-offs of: normal (ACR < 3, *n* = 86), microalbuminuria (ACR 3–30, *n* = 27), and macroalbuminuria (ACR > 30, *n* = 23). Patients with macroalbuminuria had a lower median plasma vitamin C concentration (15 [8, 28] µmol/L) compared to those with normal kidney function (29 [12, 44] µmol/L; *p* = 0.027; Figure 3A). Of note, the patients with T2DM had a higher median ACR than those with T1DM (*p* = 0.004). There were weak inverse correlations between plasma vitamin C and specific markers of renal dysfunction, including cystatin C, serum creatinine, and urine albumin (*p* < 0.01; Table 3). However, there was no significant correlation between plasma vitamin C and eGFR (*p* = 0.06), and although patients with eGFR < 60 had lower median vitamin C status (17 [11, 34] µmol/L; *n* = 44) relative to those with eGFR ≥60 (29 [12, 44] µmol/L; *n* = 92), this was only trending towards significance (*p* = 0.08; Figure 3B). The T1DM and T2DM groups had a comparable proportion of renal dysfunction (Table 1, *p* = 0.2). There were, however, differences observed between median renal function parameters (cystatin C, ACR and eGFR) between the two groups (*p* < 0.01), indicating that the patients with T2DM had more severe renal dysfunction. The significant vitamin C and renal function correlations were lost when the two groups were analysed separately.

### 3.4. Plasma Vitamin C Status Relative to Cardiometabolic Health Indices

Patients with treated hypertension had lower vitamin C status (17 [7, 38] µmol/L) than those without (30 [18, 43] µmol/L; *p* = 0.01; Figure 3C). There was a weak inverse correlation between vitamin C concentrations and diastolic blood pressure (*r* = −0.18, *p* = 0.04), but not systolic blood pressure (Table 4). This inverse correlation was stronger in the T1DM group (*r* = −0.306, *p* = 0.009) whilst there was no correlation in the T2DM group (*r* = 0.18, *p* = 0.15). Weak correlations were also observed between the vitamin C status of the cohort and blood lipids: triglycerides (*r* = −0.31, *p* = 0.0002), HDL cholesterol (*r* = 0.19, *p* = 0.03), cholesterol/HDL ratio (*r* = −0.28, *p* = 0.001), and urate (*r* = −0.27, *p* = 0.002), but not total cholesterol, LDL cholesterol, or HbA1c (Table 2). There was a significant inverse correlation between vitamin C status and body weight (*r* = −0.39, *p* < 0.0001) and BMI (*r* = −0.36, *p* < 0.0001). This inverse correlation between vitamin C status and body weight was apparent for the T1DM group (*r* = −0.291, *p* = 0.01), but not the T2DM group (*r* = −0.097, *p* = 0.4); this is likely to be due to the higher body weights and corresponding lack of spread of the vitamin C concentrations within the T2DM group.

**Figure 4:**
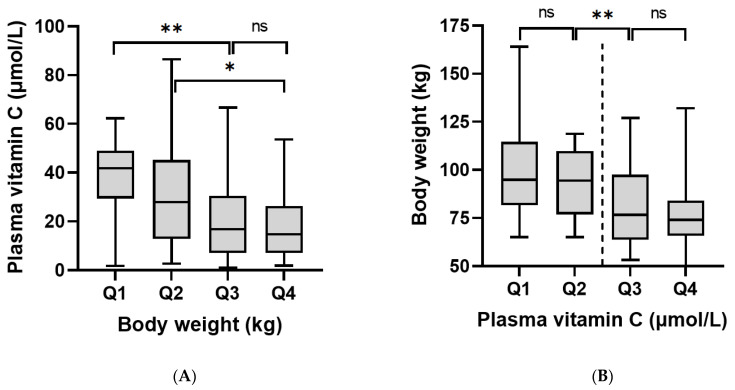
Relationships between vitamin C concentrations and body weight: (**A**) body weight quartiles: Q1 = 50–71 kg, Q2 = 72–84 kg, Q3 = 85–105 kg, Q4 = 106–164 kg (ANOVA *p* = 0.0001). (**B**) Vitamin C quartiles: Q1 = 1–12 µmol/L, Q2 = 13–22 µmol/L, Q3 = 23–42 µmol/L, Q4 = 42–87 µmol/L (ANOVA *p* < 0.001). * *p* < 0.05, ** *p* < 0.01. Dashed line indicates 23 µmol/L cut-off. Body weight was significantly different either side of this cut-off (*p* < 0.0001).

Analysis of the vitamin C data by weight quartiles indicated a significantly higher vitamin C status in the lowest weight quartile (Q1 ≤ 71 kg) relative to the top two weight quartiles (Q3 ≥ 85 kg, Q4 ≥ 106 kg; *p* < 0.0001) and between the second weight quartile (Q2 ≤ 84 kg) and the top weight quartile (Q4 ≥ 106 kg; *p* < 0.05; Figure 4A). A similar trend was observed with BMI quartiles, with significantly higher vitamin C status observed in the lowest BMI quartile (Q1 ≤ 26 kg/m^2^) relative to the other BMI quartiles (Q2 ≥ 27 kg/m^2^, Q3 ≥ 31 kg/m^2^, Q4 ≥ 36 kg/m^2^; supplemental Figure S1A). Of interest, analysis of weight data relative to vitamin C concentration quartiles revealed a significant difference in weight either side of the hypovitaminosis C cut-off (23 µmol/L; *p* = 0.0001; Figure 4B). Comparable trends were observed for BMI relative to vitamin C quartiles (*p* = 0.0004; supplemental Figure S1B). There was an inverse relationship between vitamin C status and body weight quartiles for the T1DM group (*p* = 0.04), but not the T2DM group (supplemental Figure S2). The T2DM group had higher body weight quartiles than the T1DM group. Male participants had significantly higher body weight than the female participants (92 [77, 111] kg vs. 77 [65, 97] kg, *p* < 0.001). This may have contributed to the trend in difference observed between the vitamin C status of the male and female participants (18 [8, 38] µmol/L vs. 29 [14, 44] µmol/L; *p* = 0.078).

Multivariable analysis of potential predictors of vitamin C status identified weight and diabetes type as the primary contributors (Table 5). For every increase in weight of 10 kg, there was a corresponding decrease of 2.1 µmol/L vitamin C. Having T2DM resulted in plasma vitamin C concentrations being 15 µmol/L lower than those with T1DM (*p* < 0.001). Weight explained slightly more of the variation than BMI (adjusted r: weight = 0.424, BMI = 0.409). When diabetes type was adjusted for weight, the difference between the two types was 10 µmol/L (*p* = 0.01), suggesting that weight was accounting for one third of the difference in vitamin C status between patients with T1DM and T2DM.

### 3.5. Plasma Vitamin C Status Relative to Participant Characteristics

Of the total cohort, 21 (16%) identified as Māori or Pacific Islander and 115 (84%) identified as non-Māori/Pasifika (i.e., NZ European and Asian/Other; Table 1). There were more Māori/Pasifika with T2DM than T1DM (27% vs. 5%). Plasma vitamin C status was lower in the Māori/Pasifika subgroup (16 [6, 28] µmol/L) relative to non-Māori/Pasifika (28 [12, 44] µmol/L; *p* = 0.02; supplemental Figure S3A). Body weight and BMI were higher in the Māori/Pasifika subgroup, which could have contributed to their lower vitamin C status (*p* < 0.01; supplemental Figure S3B,C). There was no correlation between socioeconomic deprivation and vitamin C status of the participants (*p* = 0.1), despite the New Zealand deprivation index being higher for the Māori/Pasifika subgroup (*p* = 0.028) and for people with T2DM relative to T1DM (Table 1), and also significant correlations between socioeconomic deprivation and body weight (*r* = 0.30, *p* = 0.0005) and BMI (*r* = 0.20, *p* = 0.02). There were also no relationships between vitamin C concentrations and smoking status of the participants (i.e., never smoked, ex-smoker, current smoker; *p* > 0.05) or between smoking status and ethnicity (*p* > 0.05) or diabetes type (Table 1). There was, however, a weak inverse correlation between vitamin C status and duration of smoking (*r* = −0.22, *p* = 0.01).

## 4. Discussion

In our cohort of patients with T1DM and T2DM who were attending outpatient clinics, we found low vitamin C status and a high proportion of hypovitaminosis C and deficiency in those with T2DM relative to T1DM. Few studies have directly compared the vitamin C status of those with T1DM and T2DM [21,22]; one of these found comparable vitamin C concentrations between the two groups while the other reported lower vitamin C status in those with T2DM. Although our two groups of participants had been recruited to have a comparable range of kidney function, they did have differences in their age and duration of diabetes; the patients with T2DM were older (median of 62 vs. 47 years) and had shorter duration of known diabetes (median of 15 vs. 20 years). This relates partly to the recruitment process and partly to the clinical–epidemiological differences between these two types of diabetes, as T1DM onset typically occurs in childhood or early to mid-adulthood, whereas T2DM is associated with obesity and typically occurs in mid to late adulthood.

There are a number of factors that can affect vitamin C status and prevalence of deficiency [28]. Renal disease resulting in impaired tubular reabsorption of filtered vitamin C and increased clearance of the vitamin has been proposed to contribute to decreased vitamin C status in people with T1DM and T2DM [17,18]. Vitamin C, being water soluble, is not stored in the body and excess is readily cleared by the kidneys, thereby maintaining saturating plasma concentrations of the vitamin [29]. The kidney tubules contain the vitamin C transporter SVCT1, which helps to maintain vitamin C homeostasis through renal reabsorption of the vitamin if plasma concentrations become inadequate [30]. Although we observed weak associations of plasma vitamin C with ACR and specific renal function markers (cystatin C, serum creatinine and urine albumin), there were only non-significant trends in associations between plasma vitamin C and eGFR. As has been observed previously [17,18], increased loss of vitamin C into urine may primarily occur in people with clinical nephropathy. Thus, urinary vitamin C concentrations may provide a clearer picture as to associations between renal function and loss of vitamin C in our cohort; this will be explored in more detail in a subsequent publication.

Previous observational research has indicated that plasma vitamin C status is associated with various cardiometabolic health parameters in people with diabetes, of which glycaemic control and obesity were the strongest correlates [13,14]. In the current study, we observed associations of vitamin C with hypertension, blood pressure and blood lipids. Hypertension is a major comorbidity of obesity and diabetes and is a significant risk factor for cardiovascular diseases. Meta-analysis of observational studies has indicated lower vitamin C concentrations in people with hypertension and inverse associations between vitamin C and both systolic and diastolic blood pressure [31]. Of note, recent meta-analyses have indicated that vitamin C supplementation in people with T2DM may improve blood pressure as well as glycaemic control and blood lipids [32,33].

In our study, the most significant vitamin C correlations were with body weight and BMI. Multivariable analysis indicated that body weight was a major independent predictor of vitamin C status in our cohort. Earlier research in a cohort with prediabetes and T2DM also indicated BMI as a significant independent predictor of plasma vitamin C, independent of dietary intake [13]. Numerous epidemiological studies around the world have found inverse correlations between plasma vitamin C status and body weight or BMI [28]. Although obesity is associated with increased inflammation and oxidative stress, which could potentially deplete vitamin C levels, one study suggested a volumetric dilution effect in people with higher body weight [34]. This premise is supported by retrospective analysis of an intervention study that indicated body weight contributed to a lack of response to vitamin C intake [35], and an intervention study in the United States that indicated lower plasma vitamin C concentrations in people with higher body weight, despite comparable vitamin C intakes [16]. Based on these findings, the authors recommended that vitamin C intakes should be based on a ‘dose per kg body weight’ or in terms of ‘desirable plasma concentrations’. Thus, people with diabetes who are overweight or obese may need higher vitamin C intakes or supplements to reach comparable vitamin C status to healthy people of normal body weight. This is an important consideration as the optimal kinetics of vitamin C-dependent enzymes are dependent on vitamin C concentrations [36].

Although body weight accounted for approximately one third of the difference in vitamin C status between those with T2DM and T1DM, other factors, such as dietary intake, are potentially contributing to the difference. It is well established that a lower fruit and vegetable intake is associated with an increased risk of developing T2DM [37]. However, following diagnosis, people with T2DM tend to increase their intake of fruits and vegetables and vitamin C [38,39], resulting in higher intakes than people without diabetes [40]. In the British National Diet and Nutrition Survey, those with diagnosed diabetes had healthier nutrient profiles than those with undiagnosed diabetes (i.e., HbA1c >6.3%, equivalent to 45 mmol/mol) [41]. We and others have previously found comparable dietary vitamin C intakes between people with T2DM and healthy controls [13,14]. A study carried out in young people with both T1DM and T2DM indicated that those with T2DM tended to have higher vitamin C intakes than those with T1DM [42]. Other research has indicated that people with T1DM and T2DM also tend to take more vitamin C supplements than the general population [43]. Although few people supplemented with vitamin C in our cohort, these were predominantly T2DM. However, it should be noted that despite dietary intake normally having a large effect on plasma vitamin C concentrations in healthy people, less of an effect is observed in people who have hypovitaminosis C and/or elevated body weight [16,28,35]. This may have important implications with regard to the setting of recommended dietary intake criteria [36].

Socioeconomic factors may play a role in vitamin C intake [44], and we did observe a higher NZ deprivation index in the T2DM group, as well as positive correlations between body weight and deprivation, although no direct correlation between vitamin C status and deprivation. Our study did show indications of ethnic differences in vitamin C status, with the group identifying as Māori/Pasifika having lower vitamin C status, although this group was relatively small (comprising 16% of the cohort). The higher body weight and BMI of the Māori/Pasifika group likely contributed to their lower vitamin C status. Other observational studies have reported lower vitamin C status in people of African descent relative to white and non-Hispanic white people [45,46], and these studies also reported inverse associations of vitamin C status with BMI. Whilst the differences in body weight are a likely contributor, other factors are possibly contributing to the lower vitamin C status of the Māori/Pasifika group. For example, traditional cooking practices have been proposed to contribute to ethnic differences in vitamin C status in Singapore [47].

Limitations of this study include the cohort being a convenience sample of participants initially recruited to represent a wide range of albuminuria and eGFR. Thus, the sample is not representative of the general diabetes population. Additionally, dietary intake data were not collected as part of the original study, precluding analysis of vitamin C intakes. Furthermore, due to the retrospective nature of the study, vitamin C analyses were carried out on stored samples, which can result in some oxidation of vitamin C over time. We did, however, treat the samples with a reducing agent to recover vitamin C that may have become oxidised during storage [48].

Our research is of relevance to the ongoing global research effort into the SARS-CoV-2 and coronavirus disease (COVID-19) pandemic. People at highest risk of becoming severely ill with COVID-19 are those with specific medical conditions, including diabetes mellitus (types 1 and 2), obesity, and chronic kidney disease [49], making people with multiple comorbidities (i.e., diabetes, obesity and/or kidney disease) potentially more susceptible to severe COVID-19. In the New Zealand context, Māori and Pasifika peoples are at increased risk for all of these risk factors [50], as well as for morbidity and mortality from severe COVID-19 [51,52]. It is interesting to note that these risk factors also contribute to hypovitaminosis C [28], as does COVID-19 itself [53]. Due to vitamin C’s well-known immune supportive roles [54], future research restoring adequate vitamin C status in people with hypovitaminosis C and infection risk factors, and exploring its effects on infection susceptibility and severity appear warranted.

## 5. Conclusions

In our cohort of patients with T1DM and T2DM, who had been selected to have a comparable proportion of renal dysfunction, we found low vitamin C status and a high prevalence of hypovitaminosis C in those with T2DM relative to T1DM. Body weight was a significant predictor for low vitamin C status and accounted for one third of the difference in vitamin C status between those with T2DM and T1DM. This finding could be due to a volumetric dilution effect and/or enhanced utilisation of the vitamin due to obesity-related inflammation and oxidative stress. Our finding suggests that obese people with diabetes may have higher requirements for vitamin C, and supplementation may be required to restore adequate vitamin C status. The doses required remain to be established.

## Figures and Tables

**Table 1:** Participant characteristics.

	Total Cohort (*n* = 136)	T1DM (*n* = 73)	T2DM (*n* = 63)
Age, years	57 (44, 67)	47 (35, 64)	62 (53, 68)
Gender, female	72 (53)	42 (58)	30 (48)
Ethnicity:			
NZ European	106 (78)	65 (89)	41 (65)
Māori or Pasifika	21 (16)	4 (5)	17 (27)
Asian or Other	9 (7)	4 (5)	5 (8)
NZ Deprivation index	3 (2, 6)	2 (1, 5)	5 (2, 7)
Diabetes duration, years	18 (10, 24)	20 (13, 33)	15 (10, 20)
Renal dysfunction ^a^	60 (44)	28 (38)	32 (51)
Treated hypertension	84 (64)	33 (46)	51 (85)
Smoking status:			
Never smoked	72 (53)	42 (57)	30 (48)
Ex-smoker	45 (33)	19 (26)	26 (41)
Current smoker	18 (13)	12 (16)	6 (10)

Values are median (Q1, Q3) or n (%). ^a^ Renal dysfunction was defined as eGFR-EPI < 60 mL/min/1.73 m^2^ or ACR > 3 g/mol.

**Table 2:** Physiological and biochemical parameters of the cohort.

	Total Cohort (*n* = 136)	T1DM (*n* = 73)	T2DM (*n* = 63)
Weight, kg	84 (71, 105)	75 (65, 85)	99 (82, 114)
BMI, kg/m^2^	30 (25, 35)	26 (23, 30)	34 (31, 38)
Diastolic BP, mmHg	80 (71, 85)	77 (70, 84)	83 (74, 88)
Systolic BP, mmHg	135 (122, 151)	131 (115, 144)	140 (129, 155)
Triglycerides, mmol/L	1.6 (1.0, 2.3)	1.2 (0.9, 1.7)	2.2 (1.6, 3.2)
HDL cholesterol, mmol/L	1.3 (1.0, 1.6)	1.5 (1.3, 1.7)	1.0 (0.9, 1.3)
Total cholesterol, mmol/L	4.7 (4.0, 5.3)	4.8 (4.2, 5.3)	4.3 (3.7, 5.1)
Cholesterol/HDL ratio	3.5 (2.9, 4.4)	3.2 (2.7, 3.8)	4.2 (3.3, 4.9)
LDL cholesterol, mmol/L	2.6 (1.9, 3.1)	2.6 (2.2, 3.1)	2.3 (1.8, 3.0)
Urate, mmol/L	0.30 (0.23, 0.38)	0.25 (0.19, 0.30)	0.35 (0.30, 0.41)
HbA1c, mmol/mol	64 (55, 75)	64 (54, 71)	65 (58, 79)
Cystatin C, mg/L	1.0 (0.8, 1.2)	0.9 (0.7, 1.0)	1.1 (0.9, 1.4)
ACR, g/mol	1.6 (0.7, 8.4)	1.2 (0.6, 3.4)	2.6 (1.0, 32.0)
eGFR, ml/min/1.73 m^2^	72 (57, 84)	76 (61, 88)	66 (51, 78)

Values are median (Q1, Q3). BMI, body mass index; BP, blood pressure; HDL, high-density lipoprotein; LDL, low-density lipoprotein; HbA1c, glycated haemoglobin; ACR, urinary albumin to creatinine ratio; eGFR, estimated glomerular filtration rate.

**Table 3:** Correlations of plasma vitamin C status with renal function parameters (*n* = 136).

Parameter	Spearman Correlation (*r*)
Plasma cystatin C	−0.25 **
Serum creatinine	−0.23 **
Urine albumin	−0.22 **
Urine creatinine	−0.07
Albumin to creatinine ratio (ACR)	−0.21 **
Estimated glomerular filtration rate (eGFR)	0.16

** correlations significant at <0.01.

**Table 4:** Correlations of plasma vitamin C status with cardiometabolic health parameters (*n* = 136).

Parameter	Spearman Correlation (*r*)
Diastolic blood pressure	−0.18 *
Systolic blood pressure	−0.05
Triglycerides	−0.31 ***
HDL cholesterol	0.19 *
Total cholesterol	−0.05
Cholesterol/HDL ratio	−0.28 ***
LDL cholesterol	−0.0005
Urate	−0.27 **
HbA1c	−0.14
Body weight	−0.39 ****
Body mass index (BMI)	−0.36 ****

* correlations significant at <0.05; ** correlations significant at <0.01; *** correlations significant at <0.001; **** correlations significant at <0.0001.

**Table 5:** Multivariable analysis of predictors of vitamin C status.

	Plasma Vitamin C (µmol/L)	
	Unadjusted	Weight Adjusted
T2DM vs. T1DM	−14.8 (−20.9, −8.8) ***	−9.7 (−16.9, −2.5) **
Weight (per 10 kg)		−2.1 (−3.7, −0.5) **

** correlations significant at <0.01; *** correlations significant at <0.001.

## Data Availability

Data is contained within the article and supplementary material.

## Supplementary Materials

The following supporting information can be downloaded at: https://www.mdpi.com/article/10.3390/antiox11020245/s1, Figure S1: Relationships between vitamin C concentrations and BMI; Figure S2: Relationships between vitamin C concentrations and body weight quartiles for (A) T1DM (ANOVA *p* = 0.04; Mann-Whitney U test of Q1 vs. Q4 *p* = 0.002) and (B) T2DM (ANOVA *p* = 0.7); Figure S3: Vitamin C, body weight and BMI relative to ethnicity. The Māori/Pasifika group (*n* = 21) was significantly different to the non-Māori/Pasifika group (*n* = 115; *p* < 0.05) for (A) vitamin C status, (B) body weight and (C) BMI.
