# Supplementary material for: Vitamin C Status in People with Types 1 and 2 Diabetes Mellitus and Varying Degrees of Renal Dysfunction: Relationship to Body Weight
Source: Antioxidants (Basel). 2022 Jan 27;11(2):245. doi: 10.3390/antiox11020245 (PMC8868094; doi:10.3390/antiox11020245)
Supplement: Supplementary file 1 [file antioxidants-11-00245-s001.zip › antioxidants-1529171-supplementary.pdf]

Supplemental Material

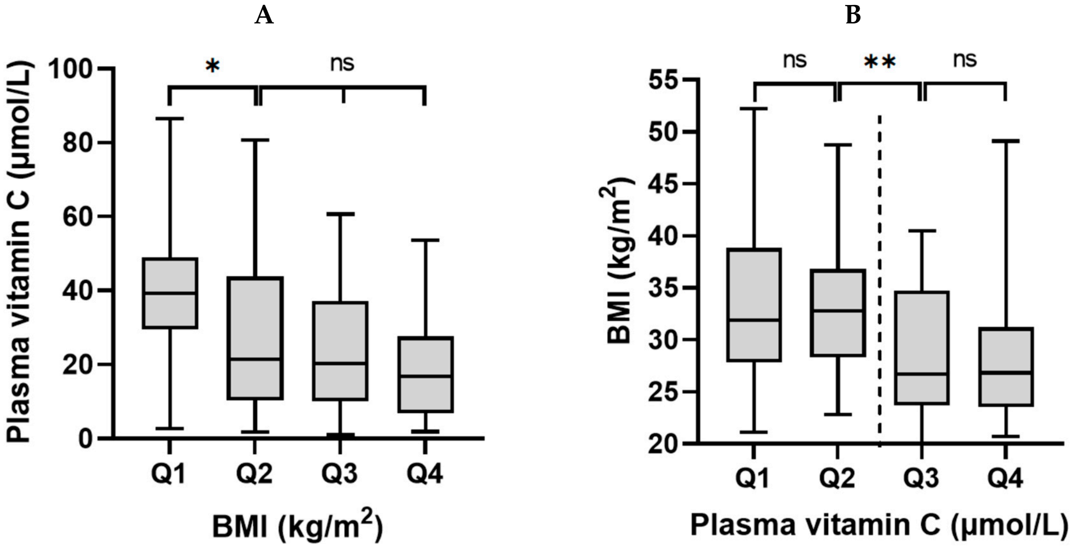

Figure S1. Relationships between vitamin C concentrations and BMI. (A) BMI quartiles: Q1 = 20-25 kg/m2, Q2 = 26-29 kg/m2, Q3 = 30-34 kg/m2, Q4 = 35-52 kg/m2 (ANOVA p = 0.0002). (B) Vitamin C quartiles: Q1 = 1-12 µmol/L, Q2 = 13-22 µmol/L, Q3 = 23-42 µmol/L, Q4 = 42-87 µmol/L (ANOVA p < 0.001). Dashed line indicates 22-23 µmol/L cutoff. Body weight was significantly different either side of this cutoff (p < 0.0004). * correlations significant at <0.05; ** correlations significant at <0.01.

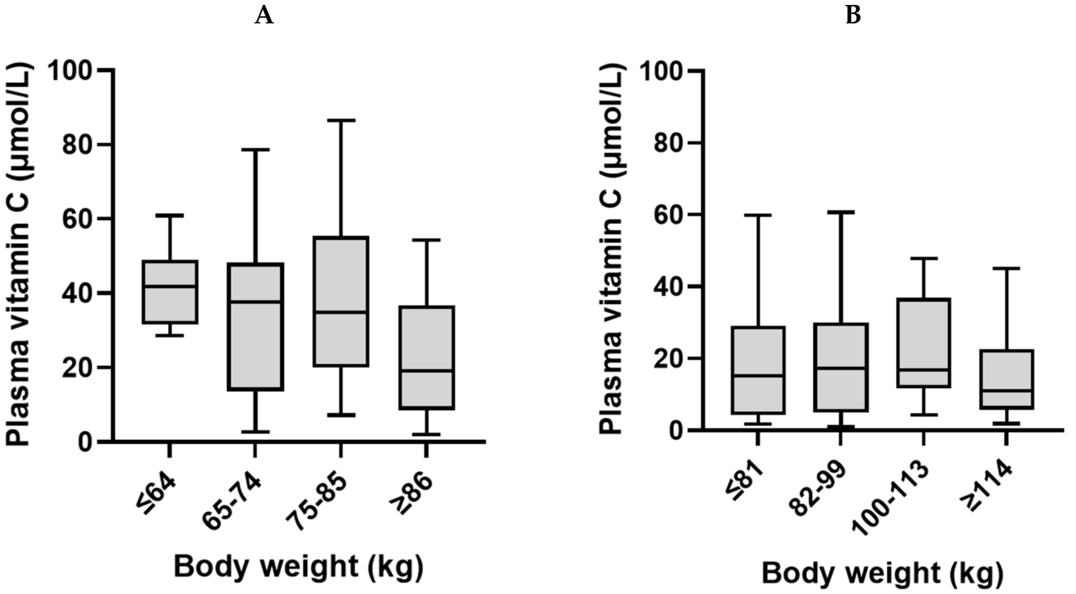

Figure S2. Relationships between vitamin C concentrations and body weight quartiles for (A) T1DM (ANOVA p = 0.04; Mann-Whitney U test of Q1 vs Q4 p = 0.002) and (B) T2DM (ANOVA p = 0.7). Note that the T1DM group had lower body weight quartiles than the T2DM group.

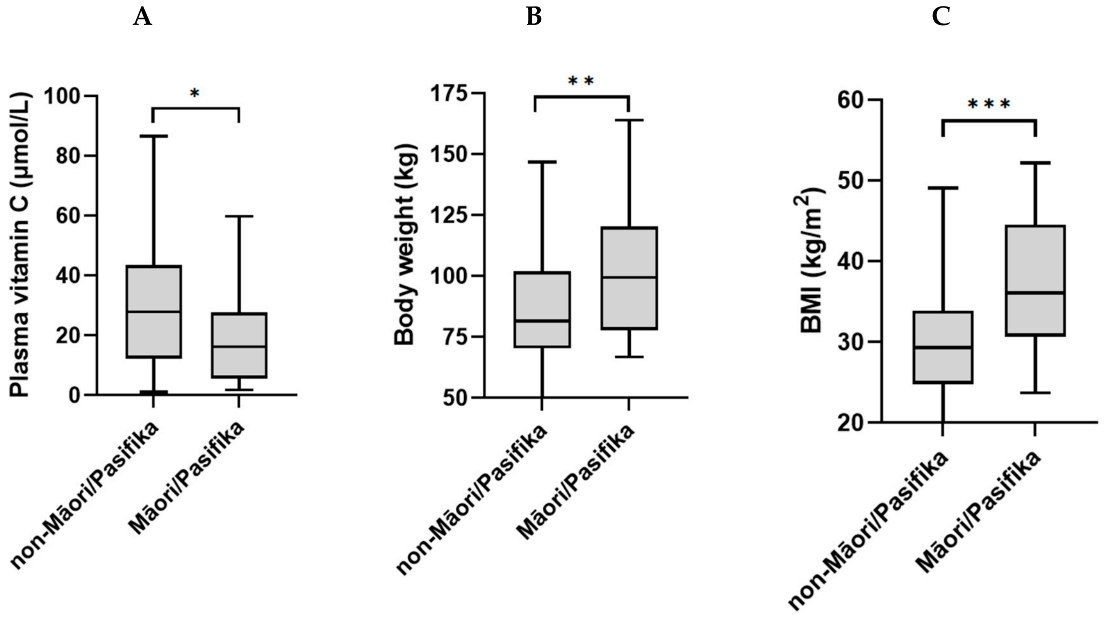

Figure S3. Vitamin C, body weight and BMI relative to ethnicity. The Māori/Pasifika group (n = 21) was significantly different to the non-Māori/Pasifika group (n = 115; $p < 0.05$) for (A) vitamin C status, (B) body weight and (C) BMI. * correlations significant at <0.05; ** correlations significant at <0.01; *** correlations significant at <0.001.
